# Clinical, Histopathological and Outcome Analysis of Five Patients With Lichenoid Eruption Following Anti-Tumor Necrosis Factor-Alpha Therapy for Ankylosing Spondylitis: Report of One Case and Review of the Literature

**DOI:** 10.7759/cureus.10598

**Published:** 2020-09-22

**Authors:** Samily Cordeiro De Oliveira, Antonio Helder Costa Vasconcelos, Emmanuel Pereira Benevides Magalhães, Fernanda Judith Vieira Corrêa, Carlos Ewerton Maia Rodrigues

**Affiliations:** 1 Medicine, Federal University of Ceará, Fortaleza, BRA; 2 Medicine, University of Fortaleza, Fortaleza, BRA; 3 Dermatology, University of Fortaleza, Fortaleza, BRA; 4 Pathology, Haroldo Juaçaba Hospital, Fortaleza, BRA; 5 Rheumatology, Federal Universiity of Ceará, Fortaleza, BRA; 6 Rheumatology, University of Fortaleza, Fortaleza, BRA

**Keywords:** anti tnf, lichen planus, ankylosing spondylitis

## Abstract

Tumor necrosis factor-alpha (TNF-ɑ) inhibitors have become the mainstay of therapy for a wide range of autoinflammatory diseases, despite concerns regarding dermatological adverse reactions. In this paper, we describe the clinical and histological findings and outcome of a case of lichenoid eruption (LE) following adalimumab therapy for ankylosing spondylitis (AS) and review four earlier reports concerning this rare clinical association. The time of onset varied considerably (three weeks to 52 months) and lesions varied within the clinical spectrum (from typical lichen planus to psoriasiform), but all had LE-compatible histology, with acanthosis, necrotic keratinocytes and lymphocytic infiltrate as hallmarks. Most patients (3/5) improved with treatment and one experienced full recovery, while in one case the lesions persisted. TNF-ɑ has been implicated in the pathogenesis of lichen planus, making it difficult to explain how TNF-ɑ antagonists can induce lichenoid reactions. The appearance of LE may in some cases justify cessation of therapy.

## Introduction

Tumor necrosis factor-alpha (TNFα) inhibitors are the mainstay of therapy for a wide range of autoimmune diseases. In patients with ankylosing spondylitis (AS), TNFα inhibitors have been shown to effectively bring symptoms under control, improve quality of life as well as reducing radiographic progression, especially with early initiation and longer duration of follow up [1].

TNF inhibitors appear to reduce radiographic progression in AS, Though relatively safe, TNFα inhibitors have been associated with various side effects, including a spectrum of skin lesions. In fact, dermatological reactions are not uncommon when TNFα inhibitors are used to treat autoimmune conditions, such as AS, rheumatoid arthritis (RA), Crohn’s disease and even psoriasis and psoriatic arthritis. Most published reports are descriptions of paradoxical psoriasiform eruptions, but other reactions are also well documented (e.g., granuloma annulare, cutaneous lupus and lupus-like syndromes) [1-3] and there is a small but increasing number of reports associating TNFα inhibitors with the onset of lichenoid eruption (LE) [1, 2].

The pathogenesis of LE is still not fully understood, with some considering it paradoxical [1, 4]. In this context, lichen planus induced by TNFα inhibitors has been observed in some types of spondyloarthritis [5, 6]. Indeed, this is supported by a study involving 252 patients with RA and 183 with spondyloarthropathy treated with TNFα inhibitors and evaluated for immune-mediated skin lesions. Only one patient in each disease group developed LE [1].

In this paper we describe the clinical presentation and histopathological findings of LE following adalimumab therapy for ankylosing spondylitis along with a review of more four cases of this rare association.

## Case presentation

A 54-year-old housewife of mixed race presented with chronic back pain, mainly on the left side, with radiation to the inferior left limb. The patient had hypertension, smoking and depression as comorbidities. The patient tested negative for human leukocyte antigen B27 (HLA-B27). Due to persistent symptoms of back pain and morning rigidity of up to two hours, associated with bilateral hip pain and increased C-reactive protein (CRP) levels, the patient was referred to the rheumatologist, and a pelvic MRI (dated 3/29/16) was requested; it showed signs of inflammatory sacroiliitis activity, and bilateral sacroiliitis was visible on a pelvic x-ray (9/15/2016). The patient was prescribed non-steroidal anti-inflammatory drugs (NSAIDs) and muscle relaxants, but the pain persisted. Four years after the onset of the inflammatory symptoms, anti-TNFɑ therapy with adalimumab was initiated.

**Figure 1:**
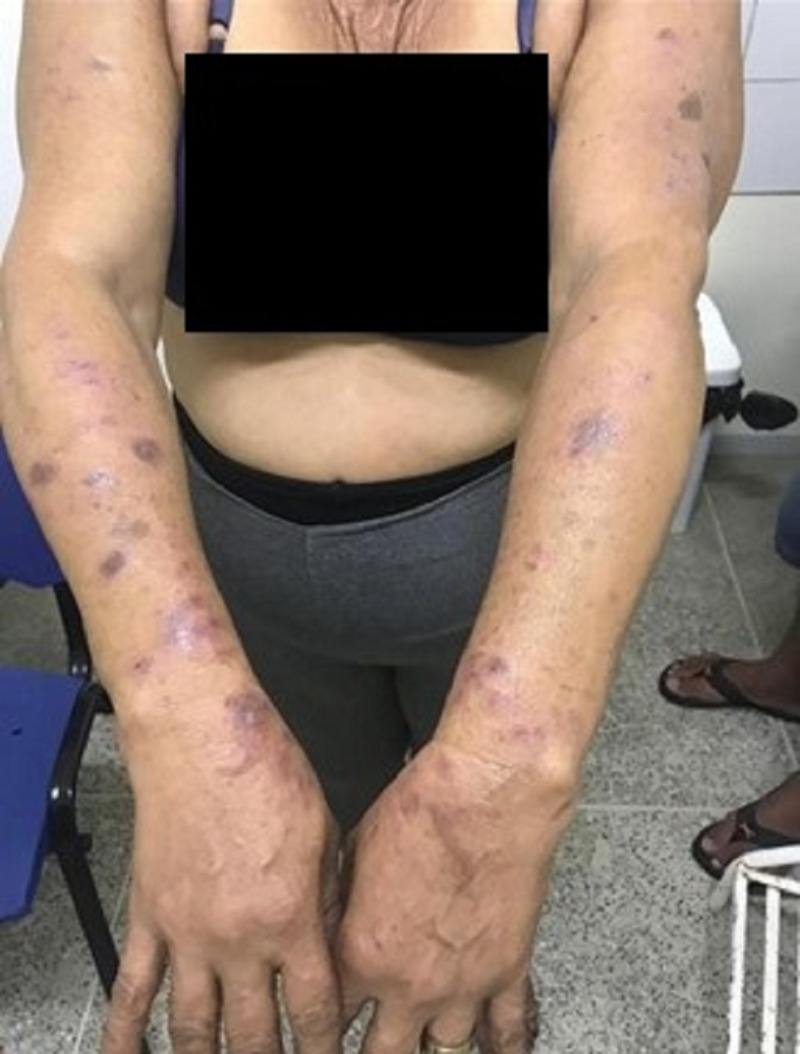
Flat polygonal papules and plaques with Wickham striae, leaving hyperchromic macules, sparing the trunk

The clinical response was good (Bath Ankylosing Disease Activity Index [BASDAI**]** reduced to <4 in a period of six months). However, after six months of treatment with adalimumab, the patient developed flat polygonal erythemato-violaceous papules and plaques on the extremities (arms and legs) with Wickham striae, but no oral lesions (Figure 1). Adalimumab was promptly discontinued and replaced with secukinumab, an interleukin (IL)-17 inhibitor. One month later, the lesions changed to pruriginous hyperchromic macules of increasing size on the left arm, and an erythematous plaque developed on the posterior left neck. Based on the diagnostic hypothesis of lichen planus, the patient was prescribed topical clobetasol, prednisone (40 mg/day, tapered) and hydroxyzine. At an encounter two months later, the pruritus had resolved and the erythematoviolaceous plaques had improved, leaving residual hyperchromic lesions, especially in sun-exposed areas. Clobetasol therapy was continued for active lesions, with the addition of hydroquinone. Dexamethasone was prescribed in order to improve the cosmetic appearance of the macules.

**Figure 2:**
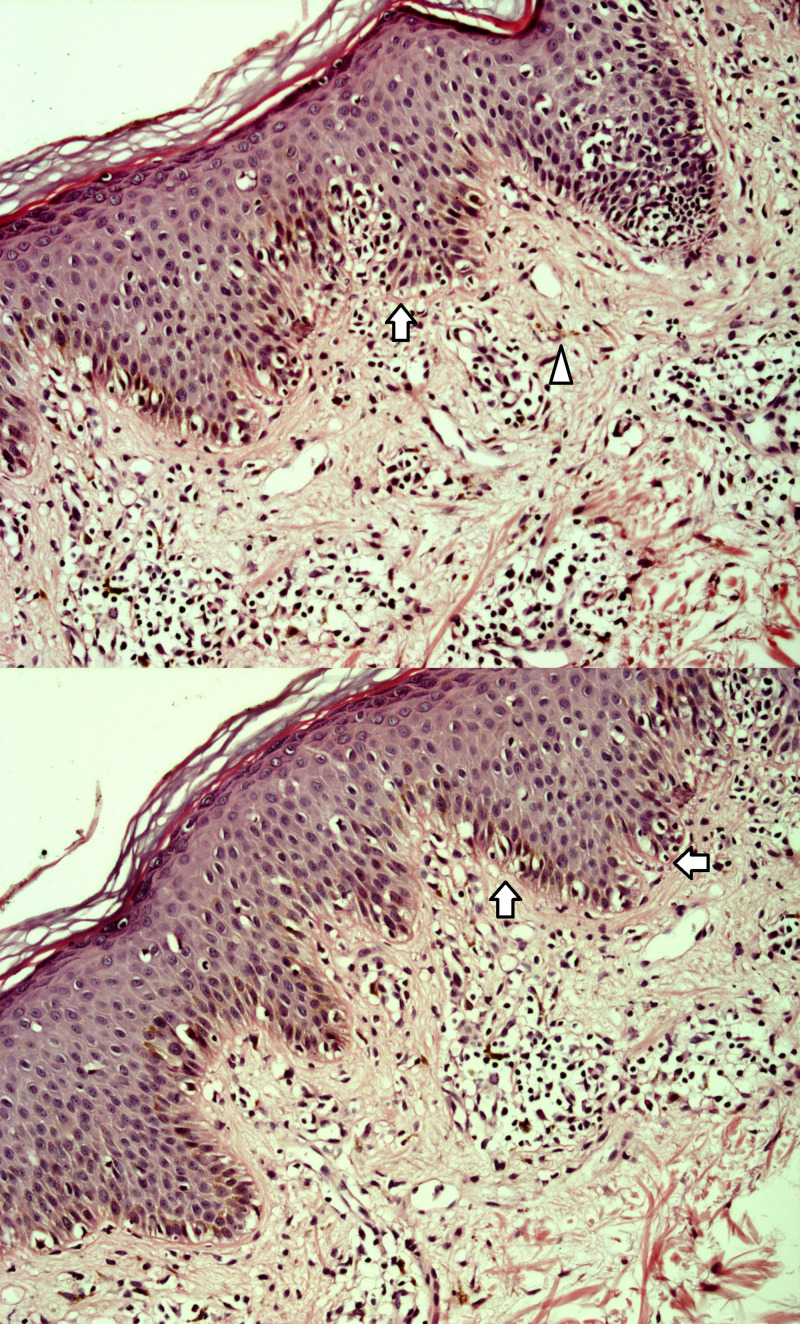
Histological skin samples showing discrete irregular acanthosis, hyperkeratosis, vacuolar degeneration of the basal layer, round bodies, band normolimphocytic inflammatory infiltrate and pigmentary effusion Arrow: bandlike chronic inflammatory infiltrate; triangle: pigmentary effusion (stain: H&E, 200x magnification)

The anatomopathological analysis confirmed the diagnosis of chronic lichenoid dermatitis (Figure 2) compatible with LE.

## Discussion

The present case of LE in a patient receiving anti-TNFɑ therapy for AS is a relevant addition to the four cases previously described [1, 7-9] (Table 1), the first of which was reported as recently as 2002 [7]. To our knowledge, this is the first article to review the clinical and histopathological findings and outcome of all known cases.

**Table 1:** Reported lichenoid reactions in patients with ankylosing spondylitis following therapy with TNFα inhibitors TNFα: tumor necrosis factor-α

Study	Patient age, y/sex	Drug	Reaction site	Clinical morphology	Time to reaction	Histopathology	Cessation of TNF inhibitor	Outcome
Exarchou et al [1] 2009	-	Infliximab	-	-	-	-	No	Treated with local corticosteroids with good results - Improvement
Vergara et al. [7] 2002	60/M	Infliximab	Cutaneous arm flexor	Erythematous papules, some polygonal in shape	3 wks	Necrotic keratinocytes, focal detachment of dermoepidermal junction, intense bandlike inflammatory infiltrate of lymphocytes, histocytes in the superficial dermis	Yes	Complete improvement - Recovery
Wendling et al. [8] 2013	47/M	Adalimumab	Legs, forearms and the perineal region + oral involvement	Pruriginous shiny, firm and reddish purple skin lesions	52 mo	Histopathological findings consistent with the diagnosis of lichen planus	Yes, change to infliximab	Skin evolution was favorable under topical and oral steroids - Improvement
Seneschal et al. [9] 2007	26/F	Infliximab	-	Plaques and widespread guttate psoriasis-like eruptions	8 mo	Parakeratosis, acanthosis, lichenoid and spongiotic pattern, necrotic keratinocytes	Yes	Persistent skin lesions in spite of cessation of infliximab for one year - Continuity
Current case	54/F	Adalimumab	Cutaneous neck and limbs	Erythematonodular plaques	6 mo	Discrete irregular acanthosis, hyperkeratosis, vacuolar degeneration of the basal layer, round bodies, band normolimphocytic inflammatory infiltrate and pigmentary effusion	Yes	Residual hyperchromic spots - Improvement

AS is commonly treated with TNFɑ inhibitors, despite the risk of adverse effects. LE is rarely reported. The five known cases of TNFɑ inhibitor-induced LE in AS patients include two males, two females and one individual of unspecified sex, with a median age of 43 years (range: 26-60). LE was caused by treatment with adalimumab or infliximab, but patients with other conditions are known to have developed LE after using etanercept and lenercept, suggesting the reaction is not limited to a specific drug but is a characteristic of the class [10, 11]. The time of onset of LE ranged from three weeks to 52 months of drug use.

Lesions were located mostly on the arms and/or legs, but two patients also reported oral involvement. Lesions were described as “erythematonodular plaques”, “erythematous papules, some polygonal in shape” [7], “pruriginous shiny, firm and reddish purple skin lesions” [8], and “plaques and widespread guttate psoriasis-like eruptions” [9]. One patient had perianal lesions. All diagnoses were confirmed by histology, but with variations within the clinical spectrum: lesions were typical of lichen planus, non-specific maculopapular morphology or even psoriasis-like [4, 9]. The histological findings were: “necrotic keratinocytes” [7, 9], “parakeratosis, acanthosis” [9], “focal detachment of the dermoepidermal junction and inflammatory infiltrate of lymphocytes” [7] and “degeneration of the basal layer, round bodies”.

In three patients (including the current case) anti-TNFα therapy was discontinued due to intense LE-related discomfort. One patient replaced the medication with another drug of the anti-TNFα class, curiously without recurrence of LE [8]. In the current case, anti-TNFα therapy was replaced with anti-IL17 therapy (secukinumab). The cutaneous outcome was mostly good: three of five patients showed improvement and one had full recovery, but in one patient the lesions persisted, even one year after infliximab was stopped. All five patients used topical corticosteroids to treat the skin lesions.

The pathogenesis of lichenoid lesions associated with the use of TNF inhibitors has not yet been fully explained. The development of this reaction is somewhat surprising since TNFα is itself involved in the pathogenesis of lichen planus, and TNFα inhibitors have been used with success to treat refractory cases, making it a paradoxical manifestation [12-13].

Some have hypothesized that LE develops due to cross-regulation between type I IFN (interferon) and TNFα in such a manner that the cytokines neutralize each other in a delicate balance. If this were the case, drug-induced inhibition of TNF-α would greatly raise the levels of type I INF, inducing the activation of T cells and dendritic cells and producing an inflammatory response favoring the appearance of lesions in genetically predisposed individuals. Thus, TNF-α inhibition may be considered both antiinflammatory and proinflammatory [13-15]. The process may also be modulated by other, as yet unidentified environmental factors.

To test this hypothesis, a blood sample was taken from a patient with LE after a period of adalimumab use and determined the IFNα concentration by enzyme-linked immunosorbent assay in a culture of supernatants from a lymphocyte stimulation test using peripheral blood mononuclear cells. In support of the theory, IFNα levels were greatly increased in the sample; nevertheless, more investigations are required to confirm this finding [14].

Patients with other (non-AS) conditions have also developed LE after anti-TNFα inhibition. Thus, a search of the literature identified 39 patients with other rheumatological diseases (RA n=14, psoriasis/psoriatic arthrithis n=12, Sjögren syndrome n=1, Crohn’s disease n=7, unspecific colitis n=1, idiopathic juvenile arthrithis n=2, and Behçet’s disease n=1) [4-7, 9-11, 14-20] (Table 2). The time from the initiation of the TNF-α blockade to the appearance of LE (when cited) ranged from three weeks to 48 months, although onset within the first two months of therapy was the most common. The tardiest reaction reported was that of a 19-year-old patient treated for juvenile idiopathic arthritis who presented itchy erythematous papular rash over the trunk and limbs after 48 months of infliximab monotherapy, with histology showing focally vacuolated dermatitis of the dermo-epidermal interface and irregular acanthosis and hypergranulosis compatible with LE. Response to steroid therapy was satisfactory and infliximab was replaced with adalimumab, without recurrence of skin lesions [6].

**Table 2:** Reported lichenoid reactions in patients with other (non-AS) conditions following therapy with TNFα inhibitors. JIA: juvenile idiopathic arthritis; IBD: inflammatory bowel disease; TNFα: tumor necrosis factor-α; AS: ankylosing spondylitis

Disease	Number of cases	Drugs	Time to reaction	Cessation of drug therapy	Outcome
Rheumatoid arthritis [7, 9-11, 14-16]	14	Etanercept Infliximab Lenercept Adalimumab	3 wks - 18 mo	Yes (10)/ No (4)	Improvement (2) / Recovery (7)
Severe psoriasis [4,15-17]	7	Etanercept Infliximab Adalimumab	5 wks - 11 mo	Yes (3)/ No(3)	Improvement (3) / Recovery (2) / Continuity (2)
Psoriatic arthritis (with or without psoriasis) [5,9,15,16]	5	Etanercept Infliximab Adalimumab	1 - 26 months	Yes (3)/ No (1)	Improvement (2) / Recovery (2)
IBD [15-20]	8	Etanercept Infliximab Certolizumab pegol Adalimumab	3 weeks - 24 months	Yes (4)/ No (3)	Improvement (2) / Recovery (4) / Continuity (1)
JIA [6,15]	2	Adalimumab, Infliximab	3 - 48 months	Yes	Improvement (1) / Recovery (1)
Behçet’s disease [15]	1	Adalimumab	12 months	No	Improvement
Sjögren syndrome [16]	1	Infliximab	Not reported	Yes	Improvement

## Conclusions

In conclusion, a growing body of evidence suggests that TNF-ɑ inhibitors can trigger a wide variety of inflammatory states, including lichen planus-like cutaneous and mucosal eruptions. Fortunately, these eruptions have good prognosis. If the patient develops LE, the decision to discontinue therapy should be made on a case-by-case basis, as evidences are scarce. In any case, adverse clinical manifestations should be detected and managed as early as possible.
